# Design, synthesis and biological evaluation of 4-bromo-N-(3,5-dimethoxyphenyl)benzamide derivatives as novel FGFR1 inhibitors for treatment of non-small cell lung cancer

**DOI:** 10.1080/14756366.2018.1460824

**Published:** 2018-05-07

**Authors:** Zixin Xie, Donghua Cheng, Lu Luo, Guoliang Shen, Suwei Pan, Yaqian Pan, Bo Chen, Xuebao Wang, Zhiguo Liu, Yuan Zhang, Faqing Ye

**Affiliations:** School of Pharmaceutical Sciences, Wenzhou Medical University, Wenzhou, Zhejiang, China

**Keywords:** Benzamide derivatives, FGFR1, inhibitors, molecular docking, NSCLC

## Abstract

A series of 4-bromo-N-(3,5-dimethoxyphenyl)benzamide derivatives were designed and synthesised as novel fibroblast growth factor receptor-1 (FGFR1) inhibitors. We found that one of the most promising compounds, **C9**, inhibited five non-small cell lung cancer (NSCLC) cell lines with FGFR1 amplification, including NCI-H520, NCI-H1581, NCI-H226, NCI-H460 and NCI-H1703. Moreover, the IC_50_ values for the compound **C9** were 1.36 ± 0.27 µM, 1.25 ± 0. 23 µM, 2.31 ± 0.41 µM, 2.14 ± 0.36 µM and 1.85 ± 0.32 µM, respectively. The compound **C9** arrested the cell cycle at the G2 phase in NSCLC cell lines. The compound **C9** also induced cellular apoptosis and inhibited the phosphorylation of FGFR1, PLCγ1 and ERK in a dose-dependent manner. In addition, molecular docking experiments showed that compound **C9** binds to FGFR1 to form six hydrogen bonds. Taken together, our data suggested that the compound **C9** represented a promising lead compound-targeting FGFR1.

## Introduction

**Figure 1.**
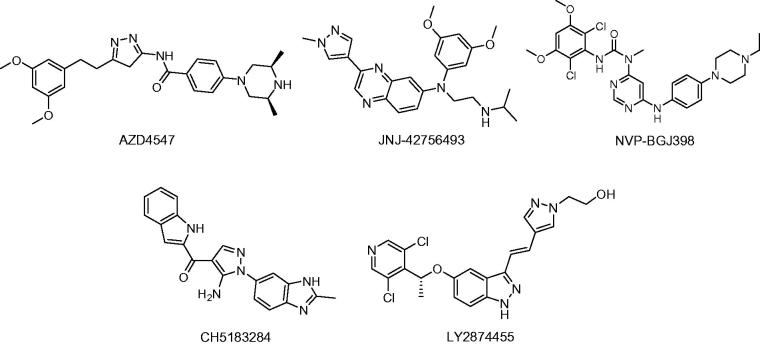
Structure of FGFR inhibitors.

Fibroblast growth factor receptors (FGFRs), a type of receptor tyrosine kinases (RTKs), are key regulators in cellular signalling that is related to cell proliferation, survival, differentiation, migration and angiogenesis^1–3^. When fibroblast growth factors (FGFs) are bound to specific receptors, they induce FGFR dimerisation and autophosphorylation, resulting in activation of downstream signalling pathways, including the MAPK and PLCγ signalling pathways^3^^,^^4^. Previous studies explained that the FGF signalling pathway plays fundamental roles not only in embryogenesis, tissue repair and wound healing, but also in tumour formation and progression^5^^,^^6^. Preclinical studies in which gene knockout and pharmaceutical inhibition of FGFRs were used, have confirmed that FGFRs are promising targets in cancer therapy^7^^,^^8^. In recent years, several small molecules that were reported to target the FGFR have entered clinical development, for example, regorafenib, ponatinib, intedanib and lenvatinib^9–11^. Due to the fact that members of the RTK family have a significant sequence homology, it was found that most of these compounds were multi-targeted, thereby leading to undesired side effects in anticancer therapies^12–14^. Thus, the development of effective FGFR inhibitors with a higher selectivity is of utmost importance for medical treatment. After the 3D structure of the FGFR protein was reported, it was easier to design selective and potent inhibitors-targeting the FGFR. Numerous representative inhibitors exist, including AZD4547^15^, NVP-BGJ398^16^, CH5183284^17^, LY2874455^18^ and JNJ-42756493^19^ (Figure 1).

FGFR1 is the main member of the FGFR family and known to be a common target of deregulation by gene amplification in several human cancers, including cancer of the breast, bladder and lung^8^. Our goal was to design a batch of novel tyrosine kinase inhibitors that simulate adenosine triphosphate (ATP) and suppress the constitutive activation of FGFR1, thereby blocking the FGFR1-driven cancer signalling pathway. According to the mechanism of action of the above-mentioned FGFR competitive inhibitors, we used the 3D structure of the FGFR1 protein as a template. The interaction area of competitive small-molecule inhibitors and proteins can be divided into four main parts^20^^,^^21^: (1) a hinge region, (2) an inward hydrophobic pocket of the hinge region (pocket 1), (3) a downward pocket of the hinge region (pocket 2) and (4) the outward extending near solvent domain. In general, the parental nucleus of all small-molecule inhibitors can interact with the amino acid residues in the hinge region through 1–3 hydrogen bonds. Based on this, the activity and selectivity can be changed by introducing appropriate pharmacophores to the side chain of the molecule. Given the fact that the patent space will become increasingly crowded, different pharmacophores may result in different pharmacological properties^22^^,^^23^. In addition, based on our previous studies on the FGFR^24^^,^^25^, we decided to develop novel pharmacophore-based inhibitors for treating FGFR1-driven cancers using a hybridisation strategy.

**Figure 2.**
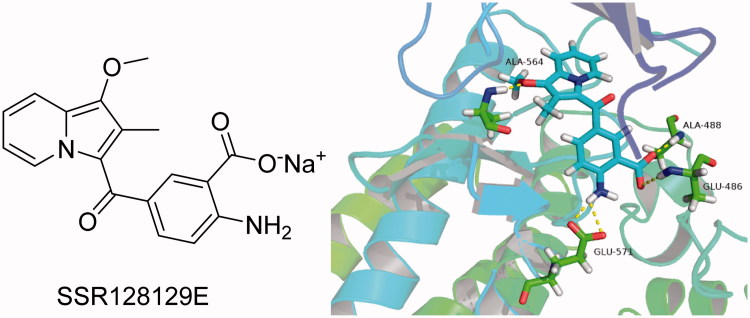
Structure of SSR128129E and molecular docking model of SSR128129E and FGFR1.

**Figure 3.**
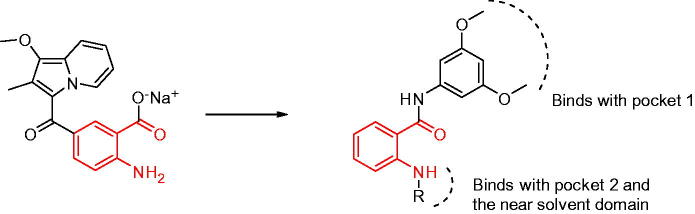
Design of novel FGFR1 inhibitors.

Bono et al.^26^ reported that compound SSR128129E is an allosteric FGFR inhibitor, the IC_50_ of FGFR1 is 1.9 µM, and there is little activity to other tyrosine kinase. In brief, SSR128129E was bound in advance to FGFR1 protein (Figure 2). The molecular docking model showed that the structure of sodium benzoate formed two hydrogen bonds with Glu486 and Ala488 in the hinge region, while the methoxyl group only formed a hydrogen-bonding interaction with Ala564 in pocket 1, and amino group formed two hydrogen-bond interactions with Glu571 in pocket 2. To develop novel small-molecule inhibitors that bind more closely to the FGFR1 kinase domain, we modified its side chain but retained the parent nucleus that strongly binds to the hinge region. Furthermore, pocket 1 is considered an important distinguishing site for small molecules to exhibit selectivity to FGFR1 enzymes, and the group that can be inserted into this hydrophobic pocket is primarily 3,5-dimethoxyphenyl^20^^,^^21^^,^^27^. Thus, as the starting scaffold, commercially available 4-bromo-2-nitrobenzoic acid was selected, to which 3,5-dimethoxybenzene was introduced at the 1-position using amide condensation to create intermediate **1**. Subsequently, intermediate **1** was reduced to produce intermediate **2**. Then, we modified the two-position to synthesise three series of compounds. As a result, a new class of FGFR1 inhibitors was obtained (Figure 3).

## Materials and methods

### Chemistry

Melting points were determined using a SGWX-4 microscopic melting point metre and were reported in an uncorrected form. ^1^H and ^13^C NMR spectra were recorded on a Bruker 600 MHz NMR spectrometer, using CDCl_3_-d_6_ or DMSO-d_6_ as solvents. Chemical shifts are expressed in ppm with TMS as internal reference. *J* values are provided in hertz. Mass spectra were recorded on a Waters Xevo TQ-S micro mass spectrometer. Reactions were monitored by thin layer chromatography (TLC) on silica gel GF-254-coated glass plates. Column chromatography was performed with 200–300 mesh silica gel.

### General procedure for preparation of intermediate 1

The following components were added to a reaction vessel: 4-bromo-2-nitrobenzoic acid (5.788 g, 0.024 mol), EDC HCl (4.518 g, 0.024 mol) and ethanol (30 ml). After the mixture was activated for 30 min at room temperature, 3,5-dimethoxyaniline (3 g, 0.020 mol) was added. The resulting solution was stirred for 5 h at 80 °C, then cooled to room temperature. Water (30 ml) was added, filtered and dried to give the intermediate **1** (5.349 g). The reaction yield was 71.6%. Physical and chemical data for intermediate 1, please refer to the supplementary material.

### General procedure for preparation of intermediate 2

Iron powder (2.292 g, 0.041 mol) and ammonium chloride (0.365 g, 0.007 mol) were added to a 100-ml flask. Next, water (40 ml) was added and the mixture was heated to at 85 °C for 10 min. Then, the temperature was gently increased, and intermediate **1** (5.349 g, 0.014 mol) was added and reacted at for 90 min at 90 °C. Subsequently, ethyl acetate (20 ml) was added, stirred for 15 min, filtered, after which the organic layer was obtained and concentrated *in vacuo*. The residue was dissolved in ethanol (20 ml), water (20 ml) was added and the residue was filtered, and dried to produce intermediate **2** (2.309 g). The reaction yield was 68.9%. Physical and chemical data for intermediate **2**, please refer to the supplementary material.

### General procedure for the preparation of A1–9

Intermediate **2** (50 mg, 0.142 mol) and different anhydride (2 ml) were added to a 25 ml flask and allowed to react at room temperature for 2 h. The organic layer was washed with saturated aqueous NaCl, dried over anhydrous Na_2_SO_4_ and concentrated *in vacuo*. The residue was purified by silica gel chromatography to produce the desired product. The purity of the target compound, please refer to the supplementary material.

### General procedure for the preparation of B1–15

A mixture of intermediate **2** (50 mg, 0.142 mol), different substituted benzoyl chlorides (200 µL) and DMAP (10 mg, 0.066 mol) as a catalyst in pyridine (2 ml) was stirred overnight at room temperature. After completion of the reaction, the mixture was concentrated *in vacuo*. The residue was added to ethanol (10 ml), refluxed for 30 min at 90 °C, cooled to room temperature, filtered and dried to result the desired compound. The purity of the target compound, please refer to the supplementary material.

### General procedure for the preparation of C1–11

A solution of 2 ml of intermediate **2** in Pridine was added substituted benzenesulfonyl chloride (0.284 mol) and DMAP as a catalyst (10 mg, 0.066 mol). After completion of the reaction, the reaction mixture was concentrated *in vacuo*. Methanol (10 ml) was added and refluxed for 30 min at 90 °C, cooled to room temperature, filtered and dried to produce the desired product. The purity of the target compound, please refer to the supplementary material.

2-Acetamido-4-bromo-N-(3,5-dimethoxyphenyl)benzamide (A1). White powder, yield: 63.7%; m.p.: 213.5–217.8 °C; ESI-MS [M + H]^+^: 392.86; ^1^H NMR (600 MHz, DMSO-d_6_) *δ* (ppm): 11.876 (s, 1H, –NH–), 9.162 (s, 1H, –NH–), 8.050 (d, *J* = 7.8 Hz, 1H, Ar–H), 8.037 (d, *J* = 7.8 Hz, 1H, Ar–H), 7.726 (s, 1H, Ar–H), 6.415 (s, 1H, Ar–H), 6.413 (s, 1H, Ar–H), 6.343 (s, 1H, Ar–H), 3.808 (s, 6H, –OCH_3_), 1.597 (s, 3H, –CH_3_). ^13^C NMR (150 MHz, DMSO-d_6_) *δ* (ppm): 169.537, 167.040, 161.140, 127.550, 125.961, 124.826, 119.045, 99.416, 55.635, 53.335, 33.136, 25.419, 14.979.

4-Bromo-N-(3,5-dimethoxyphenyl)-2-propionamidobenzamide (A2). White powder, yield: 56.3%; m.p.: 170.3–174.6 °C; ESI-MS [M + H]^+^: 406.86; ^1^H NMR (600 MHz, DMSO-d_6_) *δ* (ppm): 10.685 (s, 1H, –NH–), 8.728 (d, *J* = 1.8 Hz, 1H, Ar–H), 8.312 (s, 1H, –NH–), 7.368 (d, *J* = 8.4 Hz, 1H, Ar–H), 7.166 (s, 1H, Ar–H), 6.873 (s, 1H, Ar–H), 6.869 (s, 1H, Ar–H), 6.316 (s, 1H, Ar–H), 3.816 (s, 6H, –OCH_3_), 2.411 (d, *J* = 7.2 Hz, 2H, –CH_2_–), 1.238 (t, *J* = 15.0 Hz, 3H, –CH_3_). ^13^C NMR (150 MHz, DMSO-d_6_) *δ* (ppm): 173.226, 166.826, 161.285, 140.381, 139.170, 128.133, 127.361, 125.836, 124.635, 119.933, 99.344, 97.469, 55.616, 31.484, 19.562.

4-Bromo-2-butyramido-N-(3,5-dimethoxyphenyl)benzamide (A3). White powder, yield: 47.9%; m.p.: 137.8–141.3 °C; ESI-MS [M + Na]^+^: 443.13; ^1^H NMR (600 MHz, DMSO-d_6_) *δ* (ppm): 10.682 (s, 1H, –NH–), 8.750 (s, 1H, –NH–), 8.303 (d, *J* = 9.0 Hz, 1H, Ar–H), 7.380 (d, *J* = 9.0 Hz, 1H, Ar–H), 7.168 (s, 1H, Ar–H), 6.875 (s, 1H, Ar–H), 6.858 (s, 1H, Ar–H), 6.319 (s, 1H, Ar–H), 3.814 (s, 6H, –OCH_3_), 2.354 (t, *J* = 15.0 Hz, 2H, –CH_2_–), 1.751 (t, *J* = 15.0 Hz, 2H, –CH_2_–), 1.002 (t, *J* = 15.0 Hz, 3H, –CH_3_). ^13^C NMR (150MHz, DMSO-d_6_) *δ* (ppm): 172.503, 166.836, 161.295, 140.401, 139.131, 128.120, 127.388, 125.875, 124.662, 119.891, 99.405, 97.487, 55.622, 40.334, 18.966, 13.882.

4-Bromo-N-(3,5-dimethoxyphenyl)-2-pentanamidobenzamide (A4). White powder, yield: 51.2%; m.p.: 158.5–160.2 °C; ESI-MS [M + H]^+^: 436.91; ^1^H NMR (600 MHz, DMSO-d_6_) *δ* (ppm): 10.675 (s, 1H, –NH–), 8.722 (s, 1H, –NH–), 8.306 (s, 1H, Ar–H), 7.377 (d, *J* = 8.4 Hz, 1H, Ar–H), 7.162 (d, *J* = 8.4 Hz, 1H, Ar–H), 6.867 (s, 2H, Ar–H), 6.316 (s, 1H, Ar–H), 3.815 (s, 6H, –OCH_3_), 2.374 (t, *J* = 15.0 Hz, 2H, –CH_2_–), 1.682 (m, 2H, –CH_2_–), 1.407 (m, 2H, CH_2_–), 0.947 (t, *J* = 14.4 Hz, 3H, –CH_3_). ^13^C NMR (150 MHz, DMSO-d_6_) *δ* (ppm): 172.668, 166.830, 161.285, 140.378, 139.153, 128.132, 127.358, 125.863, 124.653, 119.920, 99.374, 97.487, 55.611, 38.162, 27.533, 22.477, 13.944.

4-Bromo-N-(3,5-dimethoxyphenyl)-2-hexanamidobenzamide (A5). White powder, yield: 46.8%; m.p.: 138.6–142.8 °C; ESI-MS [M + Na]^+^: 473.14; ^1^H NMR (600 MHz, DMSO-d_6_) *δ* (ppm): 10.699 (s, 1H, –NH–), 8.775 (d, *J* = 1.8 Hz, 1H, Ar–H), 8.169 (s, 1H, –NH–), 7.393 (d, *J* = 7.8 Hz, 1H, –Ar–H), 7.186 (s, 1H, Ar–H), 6.849 (s, 2H, Ar–H), 6.319 (s, 1H, Ar–H), 3.816 (s, 6H, –OCH_3_), 2.374 (t, *J* = 15.0 Hz, 2H, –CH_2_–), 1.728 (m, 2H, –CH_2_–), 1.362 (m, 4H, –CH_2_–), 0.916 (t, *J* = 14.4 Hz, 3H, –CH_3_). ^13^C NMR (150 MHz, DMSO-d_6_) *δ* (ppm): 172.631, 166.829, 161.306, 140.529, 139.056, 128.003, 127.475, 125.876, 124.695, 119.786, 99.363, 97.534, 55.614, 38.413, 31.470, 25.157, 22.537, 14.048.

2-Acrylamido-4-bromo-N-(3,5-dimethoxyphenyl)benzamide (A6). White powder, yield: 72.9%; m.p.: 137.2–138.9 °C; ESI-MS [M + H]^+^: 404.93; ^1^H NMR (600 MHz, DMSO-d_6_) *δ* (ppm): 9.110 (s, 1H, –NH–), 6.992 (d, *J* = 1.8 Hz, 1H, Ar–H), 6.724 (s, 1H, Ar–H), 6.149 (s, 1H, –NH–), 5.798 (s, 1H, –CH=CH_2_), 5.716 (s, 2H, Ar–H), 5.510 (d, *J* = 8.4 Hz, 1H, –Ar–H), 5.541 (s, 1H, –CH=CH_2_), 4.493 (s, 1H, Ar–H), 4.410 (s, 1H, –CH=CH_2_), 1.898 (s, 6H, –OCH_3_). ^13^C NMR (150 MHz, DMSO-d_6_) *δ* (ppm): 165.676, 162.499, 159.548, 148.201, 138.896, 137.534, 130.271, 126.867, 124.371, 123.009, 118.243, 113.250, 97.817, 96.909, 95.320, 53.561.

(E)-4-bromo-2-but-2-enamido-N-(3,5-dimethoxyphenyl)benzamide (A7). White powder, yield: 39.1%; m.p.: 186.6–189.0 °C; ESI-MS [M + H]^+^: 419.18; ^1^H NMR (600 MHz, DMSO-d_6_) *δ* (ppm): 1.025 (s, 1H, –NH–), 8.899 (s, 1H, –NH–), 8.095 (s, 1H, Ar–H), 7.425 (d, *J* = 8.4 Hz, 1H, –Ar–H), 7.233 (d, *J* = 8.4 Hz, 1H, –Ar–H), 6.839 (s, 1H, Ar–H), 6.836 (s, 1H, Ar–H), 6.445 (d, *J* = 16.8 Hz, 1H, –CH=CH–), 6.327 (s, 1H, Ar–H), 5.813 (d, *J* = 10.8 Hz, 1H, –CH=CH–), 3.821 (s, 6H, –OCH_3_), 1.269 (m, 3H, –CH_3_). ^13^C NMR (150 MHz, DMSO-d_6_) *δ* (ppm): 166.811, 164.314, 161.364, 140.711, 138.896, 132.314, 128.456, 126.413, 124.824, 119.831, 99.406, 97.590, 55.831.

4-Bromo-N-(3,5-dimethoxyphenyl)-2-(2-ethylhexanamido)benzamide (A8). White powder, yield: 68.3%; m.p.: 112.8–114.9 °C; ESI-MS [M + Na]^+^: 499.30; ^1^H NMR (600 MHz, DMSO-d_6_) *δ* (ppm): 10.825 (s, 1H, –NH–), 8.921 (d, *J* = 1.8 Hz, 1H, Ar–H), 7.904(s, 1H, –NH–), 7.445 (d, *J* = 14.4 Hz, 1H, Ar–H), 7.234 (s, 1H, Ar–H), 6.802 (s, 2H, Ar–H), 6.323 (s, 1H, Ar–H), 3.813 (s, 6H, –OCH_3_), 2.216 (m, 1H, –CH=), 2.201 (m, 2H, –CH_2_–), 1.727 (m, 2H, –CH_2_–), 1.546 (m, 4H, –CH_2_–), 1.307 (m, 3H, –CH_3_), 0.950 (m, 3H, –CH_3_). ^13^C NMR (150 MHz, DMSO-d_6_) *δ* (ppm): 175.662, 166.811, 161.364, 140.938, 138.896, 127.775, 125.959, 124.824, 119.605, 99.633, 97.590, 55.604, 51.291, 32.227, 29.958, 26.327, 22.695, 14.298, 12.256.

4-Bromo-N-(3,5-dimethoxyphenyl)-2-(2,2,2-trichloroacetamido)benzamide (A9). White powder, yield: 36.5%; m.p./: 176.0–177.5 °C; ESI-MS [M + H]^+^: 497.21; ^1^H NMR (600 MHz, DMSO-d_6_) *δ* (ppm): 12.489 (s, 1H, –NH–), 8.875 (s, 1H, –NH–), 8.158 (d, *J* = 8.4 Hz, 1H, Ar–H), 7.810 (s, 1H, Ar–H), 7.676 (d, *J* = 8.4 Hz, 1H, Ar–H), 6.544 (s, 2H, Ar–H), 6.341 (s, 1H, Ar–H), 3.799 (s, 6H, –OCH_3_). ^13^C NMR (150 MHz, DMSO-d_6_) *δ* (ppm): 160.985, 153.622, 144.931, 142.545, 142.024, 133.702, 130.973, 128.435, 118.625, 101.906, 98.858, 55.616.

4-Bromo-2-(cyclohexanecarboxamido)-N-(3,5-dimethoxyphenyl)benzamide (B1). White powder, yield: 89.7%; m.p./: 117.2–119.5 °C; ESI-MS [M + Na]^+^: 483.18; ^1^H NMR (600 MHz, DMSO-d_6_) *δ* (ppm): 10.624 (s, 1H, –NH–), 8.252 (s, 1H, –NH–), 7.800 (d, *J* = 3.0 Hz, 1H, Ar–H), 7.750 (s, 1H, Ar–H), 7.723 (d, *J* = 7.8 Hz, 1H, Ar–H), 6.712 (s, 2H, Ar–H), 6.3329 (s, 1H, Ar–H), 3.818 (s, 6H, –OCH_3_), 2.301 (m, 1H, –CH–), 1.253 (m, 10H). ^13^C NMR (150 MHz, DMSO-d_6_) *δ* (ppm): 165.676, 161.364, 140.938, 139.803, 133.676, 130.725, 128.229, 125.505, 124.597, 122.101, 120.739, 99.406, 98.044, 55.604, 29.731.

4-Bromo-N-(3,5-dimethoxyphenyl)-2-(phenylamido)benzamide (B2). Yellow powder, yield: 56.8%; m.p.: 136.4–138.4 °C; ESI-MS [M + Na]^+^: 479.40; ^1^H NMR (600 MHz, DMSO-d_6_) *δ* (ppm): 11.866 (s, 1H, –NH–), 9.134 (s, 1H, –NH–), 8.125 (d, *J* = 8.4 Hz, 1H, Ar–H), 8.051 (d, *J* = 7.8 Hz, 2H, Ar–H), 7.724 (s, 1H, Ar–H), 7.547 (d, *J* = 1.2 Hz, 2H, Ar–H), 7.533 (d, *J* = 1.2 Hz, 2H, Ar–H), 6.419 (s, 2H, Ar–H), 6.341 (s, 1H, Ar–H), 3.809 (s, 6H, –OCH_3_). ^13^C NMR (150 MHz, DMSO-d_6_) *δ* (ppm): 161.252, 156.142, 147.212, 145.717, 144.393, 132.727, 131.472, 130.390, 129.774, 128.831, 128.249, 128.032, 118.462, 100.506, 97.561, 55.574.

4-Bromo-N-(3,5-dimethoxyphenyl)-2-(2-methylphenylamido)benzamide (B3). Yellow powder, yield: 69.3%; m.p.: 125.7–130.2 °C; ESI-MS [M + Na]^+^: 492.75; ^1^H NMR (600 MHz, DMSO-d_6_) *δ* (ppm): 11.160 (s, 1H, –NH–), 9.045 (s, 1H, –NH–), 8.131 (d, *J* = 8.4 Hz, 1H, Ar–H), 7.830 (d, *J* = 7.8 Hz, 1H, Ar–H), 7.714 (d, *J* = 1.2 Hz, 1H, Ar–H), 7.477 (d, *J* = 2.4 Hz, 1H, Ar–H), 7.377 (s, 1H, Ar–H), 7.223 (t, *J* = 7.6 Hz, 2H, Ar–H), 6.327 (s, 2H, Ar–H), 6.282 (s, 1H, Ar–H), 3.769 (s, 6H, –OCH_3_), 2.497 (s, 3H, –CH_3_). ^13^C NMR (150 MHz, DMSO-d_6_) *δ* (ppm): 161.242, 132.100, 131.717, 131.619, 130.128, 129.808, 127.994, 126.314, 126.107, 100.302, 97.238, 77.262, 77.051, 55.620, 55.529, 22.353.

4-Bromo-N-(3,5-dimethoxyphenyl)-2-(2-chlorophenylamido)benzamide (B4). Yellow powder, yield: 72.1%; mp. : 105.8–108.6 °C; ESI-MS [M + Na]^+^: 512.47; ^1^H NMR (600 MHz, DMSO-d_6_) *δ* (ppm): 11.076 (s, 1H, –NH–), 10.433 (s, 1H, –NH–), 7.828 (t, *J* = 7.8 Hz, 1H, Ar–H), 7.794 (d, *J* = 1.2 Hz, 1H, Ar–H), 7.765 (d, *J* = 1.2 Hz, 2H, Ar–H), 7.607 (d, *J* = 1.2 Hz, 2H, Ar–H), 7.593 (d, *J* = 1.2 Hz, 1H, Ar–H), 6.367 (s, 2H, Ar–H), 6.236 (s, 1H, Ar–H), 3.689 (s, 6H, –OCH_3_). ^13^C NMR (150 MHz, DMSO-d_6_) *δ* (ppm): 161.085, 132.386, 132.178, 131.415, 131.203, 129.952, 128.247, 128.128, 126.982, 100.681, 97.488, 55.514.

4-Bromo-N-(3,5-dimethoxyphenyl)-2-(2-fluorophenylamido)benzamide (B5). Yellow powder, yield: 49.2%; m.p.: 303.4–306.4 °C; ESI-MS [M + H]^+^: 472.98; ^1^H NMR (600 MHz, DMSO-d_6_) *δ* (ppm): 11.430 (s, 1H, –NH–), 9.023 (s, 1H, –NH–), 7.905 (s, 1H, Ar–H), 7.895 (d, *J* = 1.2 Hz, 1H, Ar–H), 7.882 (d, *J* = 1.2 Hz, 1H, Ar–H), 7.741 (d, *J* = 1.8 Hz, 1H, Ar–H), 7.580 (d, *J* = 1.2 Hz, 1H, Ar–H), 7.566 (d, *J* = 1.2 Hz, 1H, Ar–H), 7.507 (d, *J* = 1.8 Hz, 1H, Ar–H), 6.406 (s, 2H, Ar–H), 6.309 (s, 1H, Ar–H), 3.795 (s, 6H, –OCH_3_). ^13^C NMR (150 MHz, DMSO-d_6_) *δ* (ppm): 162.497, 161.135, 154.554, 146.837, 145.022, 134.128, 132.086, 130.043, 128.227, 124.596, 118.696, 117.334, 100.766, 97.816, 55.830.

4-Bromo-N-(3,5-dimethoxyphenyl)-2-(2-methoxyphenylamido)benzamide (B6). Yellow powder, yield: 53.1%; m.p.: 209.9–211.6 °C; ESI-MS [M + Na]^+^: 507.13; ^1^H NMR (600 MHz, DMSO-d_6_) *δ* (ppm): 11.739 (s, 1H, –NH–), 10.569 (s, 1H, –NH–), 8.875 (d, *J* = 1.8 Hz, 1H, Ar–H), 8.049 (d, *J* = 1.2 Hz, 1H, Ar–H), 8.036 (d, *J* = 1.8 Hz, 1H, Ar–H), 7.723 (d, *J* = 8.4 Hz, 1H, Ar–H), 7.602 (d, *J* = 1.2 Hz, 1H, Ar–H), 7.599 (t, *J* = 6.6 Hz, 1H, Ar–H), 7.474 (t, *J* = 8.4 Hz, 1H, Ar–H), 7.083 (s, 2H, Ar–H), 6.304 (s, 1H, Ar–H), 4.018 (s, 3H, –OCH_3_), 3.735 (s, 6H, –OCH_3_). ^13^C NMR (150 MHz, DMSO-d_6_) *δ* (ppm): 166.662, 163.899, 160.923, 157.734, 140.727, 139.665, 134.775, 132.012, 130.736, 126.272, 124.997, 123.721, 121.383, 112.667, 98.637, 96.724, 56.334.

4-Bromo-N-(3,5-dimethoxyphenyl)-2-(3-chlorophenylamido)benzamide (B7). Yellow powder, yield: 57.9%; m.p.: 149.6–151.5 °C; ESI-MS [M + Na]^+^: 503.13; ^1^H NMR (600 MHz, DMSO-d_6_) *δ* (ppm): 11.788 (s, 1H, –NH–), 9.023 (s, 1H, –NH–), 8.134 (d, *J* = 8.4 Hz, 1H, Ar–H), 8.059 (s, 1H, Ar–H), 7.928 (d, *J* = 7.8 Hz, 1H, Ar–H), 7.728 (s, 2H, Ar–H), 7.573 (t, *J* = 8.4 Hz, 1H, Ar–H), 7.504 (d, *J* = 7.8 Hz, 1H, Ar–H), 7.504 (d, *J* = 7.8 Hz, 1H, Ar–H), 6.432 (s, 1H, Ar–H), 6.370 (s, 1H, Ar–H), 3.836 (s, 6H, –OCH_3_). ^13^C NMR (150 MHz, DMSO-d_6_) *δ* (ppm): 161.348, 154.545, 146.680, 144.979, 135.201, 132.862, 131.587, 130.099, 128.185, 126.272, 118.619, 100.337, 97.787, 55.271.

4-Bromo-N-(3,5-dimethoxyphenyl)-2-(3-bromophenylamido)benzamide (B8). Yellow powder, yield: 39.3%; m.p.: 139.8–145.0 °C; ESI-MS [M + Na]^+^: 555.33; ^1^H NMR (600 MHz, DMSO-d_6_) *δ* (ppm): 11.969 (s, 1H, –NH–), 9.069 (s, 1H, –NH–), 8.212 (s, 1H, Ar–H), 8.136 (d, *J* = 8.4 Hz, 1H, Ar–H), 7.971 (d, *J* = 7.8 Hz, 1H, Ar–H), 7.733 (d, *J* = 1.8 Hz, 1H, Ar–H), 7.659 (d, *J* = 7.8 Hz, 1H, Ar–H), 7.580 (d, *J* = 1.8 Hz, 1H, Ar–H), 7.566 (d, *J* = 1.2 Hz, 1H, Ar–H), 6.430 (s, 2H, Ar–H), 6.372 (s, 1H, Ar–H), 3.837 (s, 6H, –OCH_3_). ^13^C NMR (150 MHz, DMSO-d_6_) *δ* (ppm): 161.367, 154.785, 147.068, 145.706, 144.345, 137.990, 136.855, 135.493, 132.316, 131.295, 130.273, 128.117, 126.756, 123.124, 118.585, 100.542, 97.705, 55.605.

4-Bromo-N-(3,5-dimethoxyphenyl)-2-(3-nitrylphenylamido)benzamide (B9). Yellow powder, yield: 49.2%; m.p.: 223.4–225.8 °C; ESI-MS [M + Na]^+^: 523.12; ^1^H NMR (600 MHz, DMSO-d_6_) *δ* (ppm): 11.773 (s, 1H, –NH–), 10.484 (s, 1H, –NH–), 8.691 (s, 1H, Ar–H), 8.488 (s, 1H, Ar–H), 8.451 (t, *J* = 8.4 Hz, 1H, Ar–H), 8.307 (t, *J* = 7.8 Hz, 1H, Ar–H), 7.875 (d, *J* = 1.8 Hz, 1H, Ar–H), 7.862 (d, *J* = 1.8 Hz, 1H, Ar–H), 7.539 (d, *J* = 1.8 Hz, 1H, Ar–H), 6.981 (s, 2H, Ar–H), 6.279 (s, 1H, Ar–H), 3.712 (s, 6H, –OCH_3_). ^13^C NMR (150 MHz, DMSO-d_6_) *δ* (ppm): 166.889, 163.548, 160.951, 148.530, 140.685, 139.532, 136.121, 134.051, 131.363, 131.288, 127.357, 127.233, 125.621, 125.178, 124.114, 122.636, 99.772, 96.840, 55.725.

4-Bromo-N-(3,5-dimethoxyphenyl)-2-(3-methoxyphenylamido)benzamide (B10). Yellow powder, yield: 53.8%; m.p.: 139.3–143.1 °C; ESI-MS [M + Na]^+^: 507.09; ^1^H NMR (600 MHz, DMSO-d_6_) *δ* (ppm): 11.734 (s, 1H, –NH–), 8.928 (s, 1H, –NH–), 8.455 (s, 1H, Ar–H), 7.562 (d, *J* = 1.8 Hz, 1H, Ar–H), 7.555 (d, *J* = 2.4 Hz, 1H, Ar–H), 7.456 (d, *J* = 2.4 Hz, 1H, Ar–H), 7.428 (t, *J* = 17.4 Hz, 1H, Ar–H), 7.140 (d, *J* = 1.8 Hz, 2H, Ar–H), 7.094 (s, 2H, Ar–H), 6.431 (s, 1H, Ar–H), 3.921 (s, 3H, –OCH_3_), 3.845 (s, 6H, –OCH_3_). ^13^C NMR (150 MHz, DMSO-d_6_) *δ* (ppm): 167.040, 166.133, 161.593, 160.005, 155.920, 147.749, 146.160, 144.799, 140.713, 139.352, 135.720, 131.635, 130.046, 128.231, 126.188, 124.598, 120.968, 112.344, 100.769, 99.634, 55.605.

4-Bromo-N-(3,5-dimethoxyphenyl)-2-(3-methylphenylamido)benzamide (B11). Yellow powder, yield: 57.1%; m.p.: 223.6–224.9 °C; ESI-MS [M + Na]^+^: 492.2; ^1^H NMR (600 MHz, DMSO-d_6_) *δ* (ppm): 11.804 (s, 1H, –NH–), 9.127 (s, 1H, –NH–), 8.143 (d, *J* = 8.4 Hz, 1H, Ar–H), 7.901 (s, 1H, Ar–H), 7.859 (d, *J* = 7.2 Hz, 1H, Ar–H), 7.741 (d, *J* = 1.8 Hz, 1H, Ar–H), 7.549 (d, *J* = 1.8 Hz, 1H, Ar–H), 7.358 (m, 2H, Ar–H), 6.455 (s, 2H, Ar–H), 6.363 (s, 1H, Ar–H), 3.827 (s, 6H, –OCH_3_), 2.392 (s, 3H, –CH_3_). ^13^C NMR (150 MHz, DMSO-d_6_) *δ* (ppm): 161.367, 156.600, 147.522, 146.160, 144.799, 138.671, 133.678, 131.408, 129.819, 128.685, 125.734, 118.472, 100.769, 97.365, 55.605, 29.958, 21.561.

4-Bromo-N-(3,5-dimethoxyphenyl)-2–(4-methylphenylamido)benzamide (B12). Yellow powder, yield: 78.9%; m.p.: 247.5–249.8 °C; ESI-MS [M + Na]^+^: 492.31; ^1^H NMR (600 MHz, DMSO-d_6_) *δ* (ppm): 11.708 (s, 1H, –NH–), 9.054 (s, 1H, –NH–), 8.133 (d, *J* = 8.4 Hz, 1H, Ar–H), 7.954 (d, *J* = 7.8 Hz, 2H, Ar–H), 7.720 (s, 1H, Ar–H), 7.544 (d, *J* = 8.4 Hz, 1H, Ar–H), 7.249 (d, *J* = 7.8 Hz, 2H, Ar–H), 6.430 (s, 2H, Ar–H), 6.351 (s, 1H, Ar–H), 3.824 (s, 6H, –OCH_3_), 2.418 (s, 3H, –CH_3_). ^13^C NMR (150 MHz, DMSO-d_6_) *δ* (ppm): 161.367, 156.600, 147.522, 146.160, 144.572, 143.437, 131.408, 129.819, 128.231, 118.472, 100.769, 97.591, 55.605, 29.731, 21.561.

4-Bromo-N-(3,5-dimethoxyphenyl)-2-(4-chloromethylphenylamido)benzamide (B13). Yellow powder, yield: 71.3%; m.p.: 243.5–245.5 °C; ESI-MS [M + Na]+: 526.10; ^1^H NMR (600 MHz, DMSO-d_6_) *δ* (ppm): 11.694 (s, 1H, –NH–), 10.532 (s, 1H, –NH–), 9.217 (d, *J* = 4.8, 2H, Ar–H), 7.960 (d, *J* = 8.4, 1H, Ar–H), 7.857 (d, *J* = 8.4, 1H, Ar–H), 7.637 (s, 1H, Ar–H), 7.702 (d, *J* = 7.8, 1H, Ar–H), 7.514 (d, *J* = 8.4, 1H, Ar–H), 6.939 (s, 2H, Ar–H), 6.311 (s, 1H, Ar–H), 5.957 (s, 2H, –CH_2_Cl), 3.718 (s, 6H, –OCH_3_). ^13^C NMR (150 MHz, DMSO-d_6_) *δ*: 167.087, 164.962, 160.923, 146.892, 145.617, 140.302, 138.389, 135.413, 131.374, 129.673, 128.398, 126.485, 124.146, 100.125, 96.724, 63.136, 55.696.

4-Bromo-N-(3,5-dimethoxyphenyl)-2-(4-fluorophenylamido)benzamide (B14). Yellow powder, yield: 66.8%; m.p.: 152.0–154.6 °C; ESI-MS [M + Na]+: 499.19; ^1^H NMR (600 MHz, DMSO-d_6_) *δ* (ppm): 11.635 (s, 1H, –NH–), 10.483 (s, 1H, –NH–), 8.625 (d, *J* = 1.8, 1H, Ar–H), 7.986 (s, 1H, Ar–H), 7.832 (d, *J* = 8.4, 1H, Ar–H), 7.503 (d, *J* = 1.8, 2H, Ar–H), 7.489 (d, *J* = 1.8, 2H, Ar–H), 7.408 (s, 1H, Ar–H), 6.940 (s, 1H, Ar–H), 6.309 (s, 1H, Ar–H), 3.721 (s, 6H, –OCH_3_). ^13^C NMR (150 MHz, DMSO-d_6_) *δ* (ppm): 166.360, 164.771, 161.140, 140.260, 132.770, 130.500, 128.004, 126.642, 124.599, 122.784, 116.429, 101.223, 100.315, 96.911, 56.058.

4-Bromo-N-(3,5-dimethoxyphenyl)-2-(4-chlorophenylamido)benzamide (B15). Yellow powder, yield: 62.1%; m.p.: 166.4–168.5 °C; ESI-MS [M + H]^+^: 512.53; ^1^H NMR (600 MHz, DMSO-d_6_) *δ* (ppm): 11.908 (s, 1H, –NH–), 9.041 (s, 1H, –NH–), 7.987 (d, *J* = 8.4 Hz, 1H, Ar–H), 7.962 (d, *J* = 9.0 Hz, 1H, Ar–H), 7.722 (d, *J* = 1.8 Hz, 1H, Ar–H), 7.508 (m, 2H, Ar–H), 7.249 (m, 1H, Ar–H), 6.854 (s, 1H, Ar–H), 6.400 (s, 2H, Ar–H), 6.352 (s, 1H, Ar–H), 3.832 (s, 6H, –OCH_3_). ^13^C NMR (150 MHz, DMSO-d_6_) *δ* (ppm): 167.267, 164.998, 161.820, 155.466, 147.522, 145.706, 144.572, 141.848, 140.940, 139.125, 132.316, 129.593, 127.323, 126.188, 124.826, 119.833, 100.769, 97.819, 55.831.

4-Bromo-2-(cyclopropanesulfonamido)-N-(3,5-dimethoxyphenyl)benzamide (C1). White powder, yield: 77.8%; m.p.: 194.6–196.6 °C; ESI-MS [M + H]^+^: 455.03; ^1^H NMR (600 MHz, DMSO-d_6_) *δ* (ppm): 10.707 (s, 1H, –NH–), 10.334 (s, 1H, –NH–), 7.777 (t, *J* = 8.4 Hz, 2H, –C_3_H_5_), 7.682 (d, *J* = 8.4 Hz, 1H, Ar–H), 7.638 (d, *J* = 7.2 Hz, 1H, Ar–H), 7.613 (s, 2H, –C_3_H_5_), 7.541 (s, 1H, Ar–H), 7.443 (d, *J* = 7.8 Hz, 1H, Ar–H), 6.904 (s, 2H, Ar–H), 6.316 (s, 1H, Ar–H), 3.738 (s, 6H, –OCH_3_). ^13^C NMR (150 MHz, DMSO-d_6_) *δ* (ppm): 166.450, 161.135, 140.515, 134.350, 131.587, 130.099, 127.548, 125.847, 124.359, 99.700, 97.149, 55.908.

4-Bromo-N-(3,5-dimethoxyphenyl)-2-(phenylsulfonamido)benzamide (C2). White powder, yield: 81.2%; m.p.: 187.6–190.0 °C; ESI-MS [M + H]^+^: 493.02; ^1^H NMR (600 MHz, DMSO-d_6_) *δ* (ppm): 10.270 (s, 1H, –NH–), 9.739 (s, 1H, –NH–), 7.949 (d, *J* = 1.8 Hz, 1H, Ar–H), 7.718 (s, 1H, Ar–H), 7.424 (d, *J* = 8.4 Hz, 1H, Ar–H), 7.255 (m, 5H, Ar–H), 6.737 (s, 2H, Ar–H), 6.267 (s, 1H, Ar–H), 3.752 (s, 6H, –OCH_3_). ^13^C NMR (150 MHz, DMSO-d_6_) *δ* (ppm): 166.360, 161.593, 141.167, 138.898, 128.231, 126.642, 123.918, 120.060, 99.180, 97.819, 55.605, 30.866, 29.731, 6.128.

4-Bromo-N-(3,5-dimethoxyphenyl)-2-(2-fluorophenylsulfonamido)benzamide (C3). White powder, yield: 42.9%; m.p.: 195.0–196.2 °C; ESI-MS [M + H]^+^: 510.90; ^1^H NMR (600 MHz, DMSO-d_6_) *δ* (ppm): 10.524 (s, 1H, –NH–), 9.960 (s, 1H, –NH–), 7.875 (d, *J* = 1.2 Hz, 1H, Ar–H), 7.862 (d, *J* = 1.2 Hz, 1H, Ar–H), 7.850 (t, *J* = 4.2 Hz, 1H, Ar–H), 7.559 (s, 1H, Ar–H), 7.437 (d, *J* = 8.4 Hz, 1H, Ar–H), 7.389 (m, 2H, Ar–H), 6.915 (s, 2H, Ar–H), 6.322 (s, 1H, Ar–H), 3.738 (s, 6H, –OCH_3_). ^13^C NMR (150 MHz, DMSO-d_6_) *δ* (ppm): 166.450, 161.348, 160.072, 157.734, 140.090, 137.114, 131.799, 130.311, 127.760, 125.422, 123.509, 118.619, 99.487, 96.298, 56.121.

4-Bromo-2-(3-bromophenylsulfonamido)-N-(3,5-dimethoxyphenyl)benzamide (C4). White powder, yield: 58.9%; m.p.: 194.6–198.6 °C; ESI-MS [M + H]^+^: 570.82; ^1^H NMR (600 MHz, DMSO-d_6_) *δ* (ppm): 10.332 (s, 1H, –NH–), 9.960 (s, 1H, –NH–), 7.898 (t, *J* = 3.6 Hz, 1H, Ar–H), 7.816 (s, 1H, Ar–H), 7.803 (d, *J* = 1.2 Hz, 1H, Ar–H), 7.735 (d, *J* = 8.4 Hz, 1H, Ar–H), 7.649 (d, *J* = 8.4 Hz, 2H, Ar–H), 7.493 (m, 1H, Ar–H), 6.907 (s, 2H, Ar–H), 6.298 (s, 1H, Ar–H), 3.733 (s, 6H, -OCH_3_). ^13^C NMR (150 MHz, D MSO-d_6_) *δ* (ppm): 166.355, 161.170, 141.078, 137.405, 132.004, 129.627, 126.387, 125.090, 122.930, 99.813, 97.004, 55.524.

4-Bromo-2–(3-chlorophenylsulfonamido)-N-(3,5-dimethoxyphenyl)benzamide (C5). White powder, yield: 48.2%; m.p.: 196.6–199.3 °C; ESI-MS [M + H]^+^: 526.81; ^1^H NMR (600 MHz, DMSO-d_6_) *δ* (ppm): 10.351 (s, 1H, –NH–), 9.962 (s, 1H, –NH–), 7.775 (t, *J* = 3.6 Hz, 1H, Ar–H), 7.696 (d, *J* = 1.2 Hz, 2H, Ar–H), 7.683 (d, *J* = 1.2 Hz, 1H, Ar–H), 7.670 (t, *J* = 1.8 Hz, 1H, Ar–H), 7.647 (d, *J* = 8.4 Hz, 1H, Ar–H), 7.553 (m, 1H, Ar–H), 6.904 (s, 2H, Ar–H), 6.300 (s, 1H, Ar–H), 3.733 (s, 6H, –OCH_3_). ^13^C NMR (150 MHz, DMSO-d_6_) *δ* (ppm): 165.767, 160.829, 141.155, 140.454, 134.520, 133.817, 131.852, 131.476, 128.152, 126.838, 126.418, 125.924, 125.519, 125.072, 99.377, 96.680, 55.642.

4-Bromo-N-(3,5-dimethoxyphenyl)-2-(4-fluorophenylsulfonamido)benzamide (C6). White powder, yield: 49.0%; m.p.: 211.6–213.4 °C; ESI-MS [M + H]^+^: 510.90; ^1^H NMR (600 MHz, DMSO-d_6_) *δ* (ppm): 10.558 (s, 1H, –NH–), 10.292 (s, 1H, –NH–), 7.811 (d, *J* = 1.8 Hz, 2H, Ar–H), 7.802 (s, 1H, Ar–H), 7.799 (d, *J* = 1.8 Hz, 2H, Ar–H), 7.655 (d, *J* = 9.0 Hz, 2H, Ar–H), 6.883 (s, 2H, Ar–H), 6.308 (s, 1H, Ar–H), 3.735 (s, 6H, –OCH_3_). ^13^C NMR (150 MHz, DMSO-d_6_) *δ* (ppm): 166.360, 164.544, 161.140, 140.713, 135.720, 131.635, 130.500, 128.004, 125.734, 117.564, 99.861, 96.911, 56.285.

4-Bromo-N-(3,5-dimethoxyphenyl)-2-(4-iodophenylsulfonamido)benzamide (C7). White powder, yield: 57.4%; m.p.: 255.7–258.0 °C; ESI-MS [M + H]^+^: 618.88; ^1^H NMR (600 MHz, DMSO-d_6_) *δ* (ppm): 10.575 (s, 1H, –NH–), 9.980 (s, 1H, –NH–), 7.862 (d, *J* = 1.8 Hz, 2H, Ar–H), 7.848 (s, 1H, Ar–H), 7.659 (d, *J* = 1.8 Hz, 2H, Ar–H), 7.471 (d, *J* = 9.0 Hz, 2H, Ar–H), 6.890 (s, 2H, Ar–H), 6.303 (s, 1H, Ar–H), 3.740 (s, 6H, –OCH_3_). ^13^C NMR (150 MHz, DMSO-d_6_) *δ* (ppm): 166.038, 160.972, 140.512, 138.897, 131.562, 128.884, 125.447, 99.550, 99.330, 96.949, 55.807.

4-Bromo-N-(3,5-dimethoxyphenyl)-2-(4-methoxyphenylsulfonamido)benzamide (C8). White powder, yield: 55.1%; m.p.: 197.4–200.2 °C; ESI-MS [M + H]^+^: 528.91; ^1^H NMR (600 MHz, DMSO-d_6_) *δ* (ppm): 10.491 (s, 1H, –NH–), 10.273 (s, 1H, –NH–), 7.669 (d, *J* = 1.8 Hz, 2H, Ar–H), 7.658 (d, *J* = 2.4 Hz, 1H, Ar–H), 7.528 (d, *J* = 2.4 Hz, 2H, Ar–H), 7.000 (d, *J* = 2.4 Hz, 1H, Ar–H), 6.988 (d, *J* = 1.8 Hz, 1H, Ar–H), 6.895 (s, 2H, Ar–H), 6.315 (s, 1H, Ar–H), 3.736 (s, 6H, –OCH_3_), 2.499 (s, 3H, –OCH_3_). ^13^C NMR (150 MHz, DMSO-d_6_) *δ* (ppm): 166.378, 163.473, 160.959, 140.399, 132.054, 131.457, 130.523, 129.631, 127.527, 125.684, 124.527, 124.313, 115.206, 99.736, 96.967, 56.204.

4-Bromo-2-(4-tert-butylphenylsulfonamido)-N-(3,5-dimethoxyph-enyl)benzamide (C9). White powder, yield: 42.9%; m.p.: 222.3–225.4 °C; ESI-MS [M + H]^+^: 548.96; ^1^H NMR (600 MHz, DMSO-d_6_) *δ* (ppm): 10.584 (s, 1H, –NH–), 10.269 (s, 1H, –NH–), 7.687 (s, 1H, Ar–H), 7.679 (d, *J* = 2.4 Hz, 1H, Ar–H), 7.664 (d, *J* = 2.4 Hz, 1H, Ar–H), 7.517 (d, *J* = 1.2 Hz, 2H, Ar–H), 7.503 (s, 1H, Ar–H), 7.467 (d, *J* = 8.4 Hz, 1H, Ar–H), 6.904 (s, 2H, Ar–H), 6.310 (s, 1H, Ar–H), 3.730 (s, 6H, –OCH_3_), 1.193 (s, 9H, –C(CH_3_)_3_). ^13^C NMR (150 MHz, DMSO-d_6_) *δ* (ppm): 166.305, 160.966, 157.212, 140.394, 138.889, 136.414, 131.481, 127.669, 127.213, 126.893, 125.631, 124.768, 124.601, 99.705, 99.323, 96.927, 55.791, 35.431, 31.092.

4-Bromo-2-(2,4-difluorophenylsulfonamido)-N-(3,5-dimethoxyph-enyl)benzamide (C10). White powder, yield: 59.5%; m.p.: 235.7–239.6 °C; ESI-MS [M + H]^+^: 528.91; ^1^H NMR (600 MHz, DMSO-d_6_) *δ* (ppm): 10.512 (s, 1H, –NH–), 9.961 (s, 1H, –NH–), 7.908 (s, 1H, Ar–H), 7.897 (d, *J* = 2.4 Hz, 1H, Ar–H), 7.883 (d, *J* = 2.4 Hz, 1H, Ar–H), 7.869 (s, 1H, Ar–H), 7.556 (d, *J* = 1.8 Hz, 1H, Ar–H), 7.407 (d, *J* = 2.4 Hz, 1H, Ar–H), 6.897 (s, 2H, Ar–H), 6.311 (s, 1H, Ar–H), 3.733 (s, 6H, –OCH_3_). ^13^C NMR (150 MHz, DMSO-d_6_) *δ* (ppm): 166.360, 160.912, 140.713, 133.224, 131.635, 125.507, 113.252, 107.124, 99.634, 96.911, 55.831.

4-Bromo-2-(2,4-dichlorophenylsulfonamido)-N-(3,5-dimethoxyphenyl)benzamide (C11). White powder, yield: 50.7%; m.p.: 250.3–255.6 °C; ESI-MS [M + H]^+^: 560.95; ^1^H NMR (600 MHz, DMSO-d_6_) *δ* (ppm): 10.582 (s, 1H, –NH–), 9.952 (s, 1H, –NH–), 8.053 (d, *J* = 8.4 Hz, 1H, Ar–H), 7.771 (d, *J* = 1.8 Hz, 1H, Ar–H), 7.743 (d, *J* = 8.4 Hz, 1H, Ar–H), 7.627 (d, *J* = 2.4 Hz, 1H, Ar–H), 7.612 (d, *J* = 1.8 Hz, 1H, Ar–H), 7.521 (d, *J* = 1.8 Hz, 1H, Ar–H), 6.914 (s, 2H, Ar–H), 6.318 (s, 1H, Ar–H), 3.738 (s, 6H, –OCH_3_). ^13^C NMR (150 MHz, DMSO-d_6_) *δ* (ppm): 166.263, 160.994, 140.364, 139.748, 133.243, 132.575, 132.187, 131.664, 128.640, 125.895, 123.646, 123.372, 99.668, 96.991, 55.790.

### Biological evaluation

#### Cell lines and reagents

This study selected five non-small cell lung cancer (NSCLC) cell lines harbouring FGFR1 amplification, including NCI-H520 (squamous), NCI-H1581 (large cell carcinoma), NCI-H226 (squamous), NCI-H460 (large-cell carcinoma) and NCI-H1703 (squamous). Four EGFR addicted cell lines were selected, including A549 (WT EGFR/k-RAS dependent), PC-9 (EGFR del E746_A750), A431 (overexpressed WT EGFR) and HCC827 (EGFR dependent/WT k-RAS). There is also a normal lung cell (BEAS-2B cell). These cell lines were all purchased from the library of the Chinese Academy of Sciences. NCI-H520, NCI-H1581, NCI-H226, NCI-H460, NCI-H1703, A549 and HCC827 cells were grown in RPMI 1640 media, supplemented with 10% of calf serum (GIBCO) and penicillin/ streptomycin (GIBCO). PC-9, A431 and BEAS-2B cells were grown in DMEM (GIBCO) containing 10% of FBS (GIBCO). Cells were maintained at 37 °C in a 5% of CO_2_ incubator.

#### Antiproliferative activity *in vitro*

NCI-H520, NCI-H1581, NCI-H226, NCI-H460, NCI-H1703, A549, PC-9, A431, HCC827 and BEAS-2B cells were cultured until cells were in the logarithmic phase, after which 5000–10 000 cells/well were seeded in 96-well plates in growth medium containing 10% of FBS, and grown overnight. Cells were treated with various concentrations of each compound in triplicate and cultured in 10% of FBS medium for 48 h. Cells treated with dimethyl sulfoxide (DMSO) served as the control. Next, tetrazolium dye (MTT) solution (5 mg/mL, 20 µL/well) was added and incubated for 4 h. After removing the supernatant, the generated formazan crystals were dissolved in 150 µL of DMSO and the absorbance was read using a spectrophotometer at a wavelength of 490 nm using an enzyme-linked immunosorbent assay plate reader. The data were calculated using GraphPad Prism version 5.0 software (GraphPad, San Diego, CA). The IC_50_ values were calculated and fitted using a non-linear regression model with a sigmoidal dose response.

#### Western blot analysis

NCI-H226, NCI-H520 and NCI-H1581 cells were treated with the indicated dose of **C9** for 2 h and lysed in 1 × sodium dodecyl sulphate (SDS) sample buffer. Equivalent amounts of proteins were loaded into 10% of SDS − PAGE gels and transferred onto nitrocellulose membranes. An ECL kit (Bio-Rad, Hercules, CA) was used visualise the immunoreactive bands, and the results were analysed using ImageJ software (National Institute of Health, MD). Antibodies directed against phospho-FGFR1, FGFR1, phospho-PLCγ1, PLCγ1, phospho-ERK and ERK (Cell Signalling Technology, Danvers, MA) were used for Western blot analysis. An antibody directed against GAPDH was used as a loading control (Bioword Technology, Louis Park, MN)^28–30^.

#### Cell apoptosis assay

NCI-H226 and NCI-H1581 (2 × 10^5^) cells were seeded into six-well plates. After overnight culture, fresh growth media containing compound **C9** (5, 10 and 20 µM) as well as a positive drug SSR128129E (20 µM) were added. RPMI 1640 medium containing 1‰ DMSO was used as a control. After culturing for 48 h, the growth medium was collected and cells were trypsinised and collected in the corresponding medium. After centrifugation at 4 °C for 5 min, the supernatant was removed and the cells were washed twice with pre-cold PBS. One hundred microliter of 1 × binding buffer, 5 µL of PI (Becton Dickinson, Franklin Lakes, NJ) and 5 µL of FITC-labelled Annexin-V (FITC-Annexin V, Becton Dickinson) were added. Cells were then gently vortexed and incubated for 15 min at 25 °C in the dark. Next, 1 × binding buffer was used to a final volume of to 500 µL. Cells were then stained with PI and Annexin-V was used as a positive control. Cells underwent flow cytometry using a BD Accuri™ C6 flow cytometer (Becton Dickinson) and the data were processed using FlowJo 7.6.1 software (Brea, CA, USA).

#### *In vitro* cell cycle effects

NCI-H1581 and NCI-H1703 (2 × 10^5^) cells were seeded into six-well plates and cultured overnight. Fresh growth media containing **C9** (2.5, 5.0, 7.5 and 10 µM) as well as a positive drug SSR128129E (10 µM) were added. Medium containing 1‰ DMSO was used as a control. After culturing for 24 h, the growth medium was collected and the cells were trypsinised and collected in the corresponding medium. After centrifugation at 4 °C for 5 min, the supernatant was removed and the cells were washed twice with pre-cold PBS. Cells were fixed in 70% of cold ethanol, incubated overnight at –20 °C, and stained with PI/RNase staining buffer (BD Pharmingen, San Jose, CA). Flow cytometry was performed using a BD Accuri™ C6 flow cytometer (Becton Dickinson) and the data were processed using FlowJo 7.6.1 software.

#### *In vitro* kinase activity

The 1× kinase reaction buffer and termination solution were prepared in advance. A total of 10 µL peptide substrate (2.5×) was added to 5 µL of (5×) compound liquid, incubated for 10 min at room temperature, and added to an additional 10 µL of peptide substrate (2.5×). The mixture was allowed to react at 28 °C. After a period of time, the termination solution was added to terminate the reaction. Data were collected through the Caliper. The inhibition rate of kinase activity% = (max – conversion)/(max – min) × 100. The “max” represented DMSO without compound, “min” represented low control.

#### Molecular docking model

Molecular docking was found using the ligand-fitting module of Autodock (version 4.2.6) (Olson Laboratory, La Jolla, CA, USA). In brief, the crystal structure of FGFR1 was downloaded from the Protein Data Bank (PDB) database, and the PDB code was 4V05. The molecular models were created using software PyMol^31^^,^^32^.

#### Statistical analysis

All experiments were performed in triplicate (*n* = 3), and the data are presented as the mean ± SEM. Statistical analyses were performed using GraphPad Pro Prism 5.0 software (GraphPad, San Diego, CA). The Student’s *t*-test and a two-way ANOVA were used to analyse the differences between treatment groups. A *p* value of <.05 was considered statistically significant.

## Results and discussion

### Chemistry

**Scheme 1.**
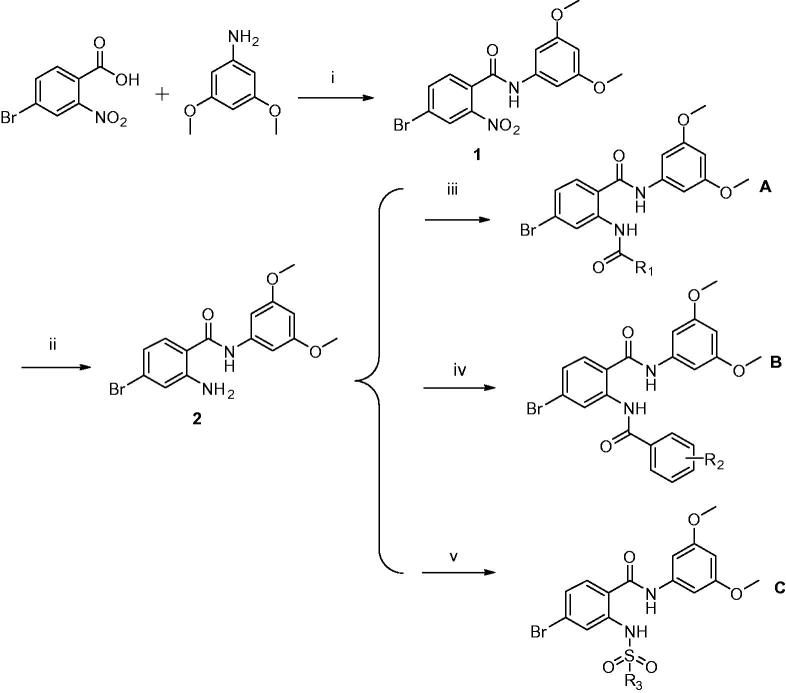
Synthetic routes of compounds.

As outlined in Scheme 1, we designed and synthesised three series of compounds (Table 1). In the A series, we primarily aimed to increase the hydrophobic effects by introducing chain alkanes. In the B series, our goal is to increase the binding to protein-binding sites and increase the space utilisation of compounds in hydrophobic pocket 2 and near solvent domains, so we substituted them with benzene rings to create π–π interactions. Finally, in the C series, we added a sulphur oxygen double bond to increase the formation of hydrogen bonds with other amino acid residues of FGFR1, thereby increasing the adhesion strength of the compounds with FGFR1 protein.

**Table 1.** Chemical structures of target compounds.

Compounds	Structure	Compounds	Structure	Compounds	Structure



Reagents and conditions: (1) ethanol, EDC**·**HCl, DMAP, 80 °C; (2) H_2_O, Fe, NH_4_Cl, 90 °C; (3) different anhydride, rt; (4) different benzoyl chloride, DMAP, pyridine, rt; (5) different benzene sulfonyl chloride, DMAP, pyridine, rt.

### Biological evaluation

#### Antiproliferative effect of synthetic compounds

Antiproliferative activities of the synthesised compounds were evaluated against five NSCLC cell lines harbouring FGFR1 amplification (NCI-H520, NCI-H1581, NCI-H226, NCI-H460 and NCI-H1703) and compared with those of DMSO and SSR128129E. The concentration of all compounds was 10 µM. The MTT assay was performed using a standard protocol. In Figure 4(A), compounds were tested for their inhibitory effect on NCI-H520 cells and compounds **A9**, **B13**, **C2**, **C5**, **C6**, **C7** and **C9** were found to have greater than 50% inhibition. Moreover, compounds **A9**, **B13** and **C9** were more active than compound SSR128129E; Figure 4(B) shows the results of compounds treated with NCI-H1581 cells. The inhibitory effect of **A9**, **B3**, **B13** and **C9** was above 50%, and the activity of **C9** was better than that of SSR128129E. Figure 4(C) shows the antiproliferative effect of the compounds on NCI-H226 cells. The inhibitory rates of all the compounds in the C series and compound **B9** were over 50%, better than the lead compound SSR128129E. Similarly, Figure 4(D) shows the antiproliferative effect of the compounds on NCI-H460 cells. The inhibitory rates of **B9**, **C3** and **C9** were greater than 50%. Moreover, the activity of **B9**, **C3** and **C9** was better than that of SSR128129E. Finally, Figure 4(E) shows the inhibitory effect of the compounds on NCI-H1703 cells. The inhibitory rates of **C4**, **C5** and **C9** were greater than 50%. In addition, **C4**, **C5** and **C9** are more active than SSR128129E.

Figure 4.Inhibition rate of NCI-H520 cell (A), NCI-H1581 cell (B), NCI-H226 cell (C), NCI-H460 cell (D) and NCI-H1703 cell (E) treated with 10 μM of target compounds and the control groups (*p < .05).

**Figure:**
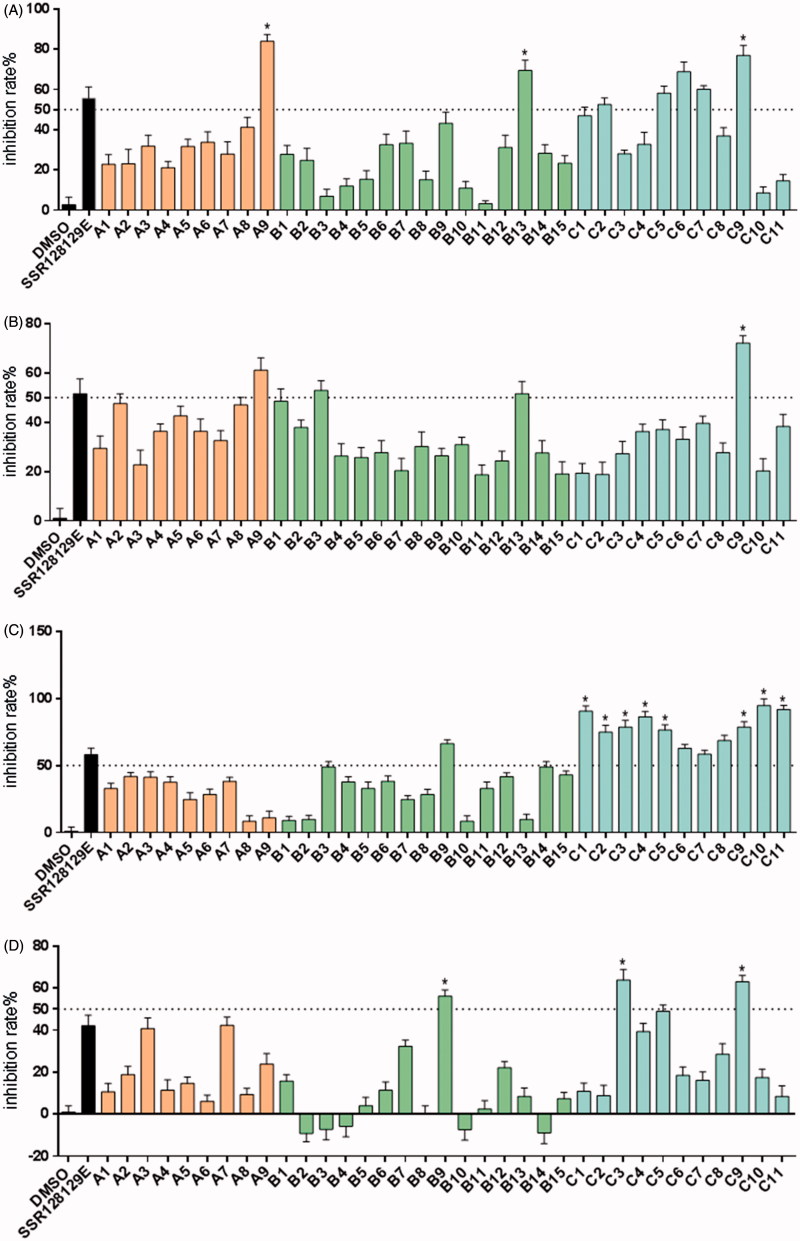

**Figure:**
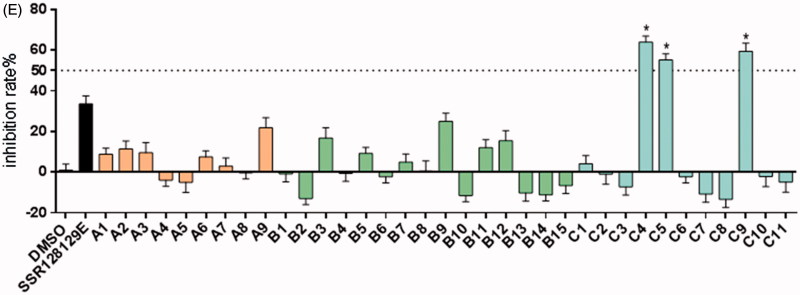

#### The IC_50_ of 10 compounds used to treat different cell lines

A total of 10 compounds with a suppression rate of at least two cell lines greater than 50% were chosen, including **A9**, **B9**, **B13**, **C2**, **C3**, **C4**, **C5**, **C6**, **C7** and **C9**. Seven concentrations (30, 20, 10, 1.0, 0.50, 0.10 and 0.01 µM) were set for these compounds. Five cell lines, including NCI-H520, NCI-H1581, NCI-H226, NCI-H460 and NCI-H1703, were treated with various concentrations of each compound, respectively. The optical density (OD) value was obtained and the inhibition rate was calculated, and the IC_50_ of the different compounds was calculated by GraphPad Prism 5 software. The results are presented in Table 2, and indicate that compound **C9** affected the five cell lines with an IC_50_ of 1.36 ± 0.27 µM, 1.25 ± 0.23 µM, 2.31 ± 0.41 µM, 2.14 ± 0.36 µM and 1.85 ± 0.32 µM, respectively, which is better than that of SSR128129E obviously. Furthermore, the toxic effect of the compounds on BEAS-2B cells showed that 30 µM of the target compound had no toxic side effects on normal lung cells (see supplementary material). Therefore, compound **C9** was chosen for further mechanistic studies.

**Table 2.** IC_50_ of NCI-H520, NCI-H1581, NCI-H226, NCI-H460 and NCI-H1703 cells treated with different compounds.

Compound	NCI-H520 (μM)	NCI-H1581 (μM)	NCI-H226 (μM)	NCI-H460 (μM)	NCI-H1703 (μM)
**A9**	2.01 ± 0.21	1.34 ± 0.28	>30	>30	>30
**B9**	>30	>30	1.98 ± 0.31	3.87 ± 0.43	>30
**B13**	1.54 ± 0.37	1.90 ± 0.47	>30	>30	>30
**C2**	4.19 ± 0.69	>30	3.21 ± 0.54	>30	>30
**C3**	>30	>30	4.13 ± 0.59	5.89 ± 0.76	>30
**C4**	12.56 ± 2.25	14.11 ± 2.81	2.28 ± 0.37	>30	3.89 ± 0.55
**C5**	5.95 ± 0.62	13.59 ± 1.62	2.49 ± 0.42	>30	4.56 ± 0.77
**C6**	6.79 ± 0.75	13.15 ± 1.60	2.42 ± 0.44	>30	>30
**C7**	4.52 ± 0.68	>30	3.16 ± 0.57	>30	>30
**C9**	1.36 ± 0.27	1.25 ± 0. 23	2.31 ± 0.41	2.14 ± 0.36	1.85 ± 0.32
SSR128129E	19.63 ± 3.43	23.98 ± 4.63	22.34 ± 4.48	29.76 ± 4.85	25.75 ± 4.72

The value “>30” indicates that no inhibitory effect at 30 μM compound concentration.

#### Structure–activity relationship analysis

In combination with the inhibitory effect of all compounds on five cell lines, we found that compounds in the A series have no effect on the activity as the alkane chain increases. Among them, compound **A9**, which contains more electronegative groups, shows more excellent inhibitory activity. Compounds **B9** and **B13** are the most prominent compounds in the B series, they are the nitro group and the p-chloromethyl group on the benzene ring, respectively. It can be concluded from the other compounds that the stronger the electronegativity of the substituent groups on the benzene ring, the poorer the activity. Due to the introduction of sulfonyl groups, we found a large number of more effective compounds in the C series of compounds, while there is a negative correlation between the electronegativity and activity of many compounds, such as compounds **C8–C11**. The increase of the electronegativity of benzene ring decreases the activity obviously. The activity of p-tert-butyl substituted compound **C9** was the best among all the target compounds.

#### Inhibitory effects of C9 on FGFR1-mediated signalling pathways in cancer cells

**Figure 5.**
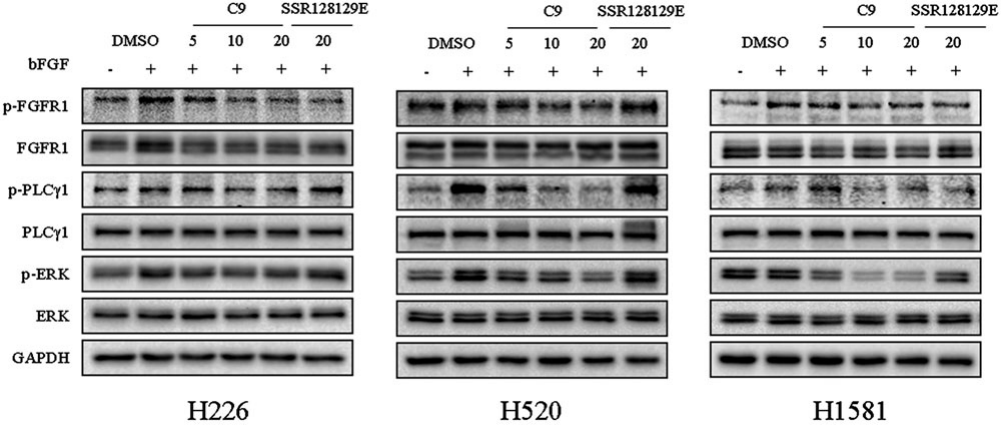
Inhibitory effect of **C9** on FGFR1 and its downstream ERK and PLCγ1 phosphorylation levels in NCI-H226, NCI-H520 and NCI-H1581 cells; *p < .05, **p < .01 compared to the control groups.

To evaluate the inhibitory effect on FGFR1 phosphorylation and the downstream signalling transduction, Western blot analysis of compound **C9** in NCI-H226, NCI-H520 and NCI-H1581 cells was performed. The data, presented in Figure 5, indicated that compound **C9** inhibited the phosphorylation of FGFR1, PLCγ1 and ERK in a dose-dependent manner. In addition, the effect of 20 µM of **C9** on the phosphorylation of FGFR1, PLCγ1 and ERK was better compared to that of SSR128129E.

#### Compound C9 induces cellular apoptosis

**Figure 6.**
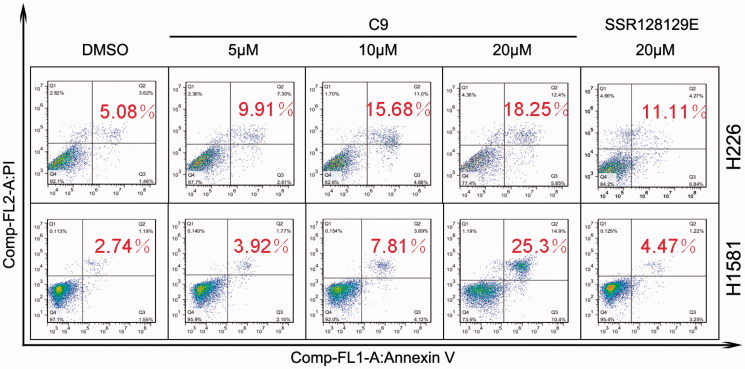
Effects of compound **C9** on apoptosis in NCI-H226 and NCI-H1581 cells compared to the positive control compound SSR128129E.

The pro-apoptotic effects of compound **C9** were examined by an Annexin V/propidium iodide (PI) assay (Figure 6). NCI-H226 and NCI-H1581 cells were treated with compound **C9** for 48 h. We determined that a concentration of 10 µM of **C9** exhibited a significant effect on the overall apoptosis rate. Compared to SSR128129E, compound **C9** was found to promote apoptosis in a dose-dependent manner.

#### Compound C9 affects the cell cycle

**Figure 7.**
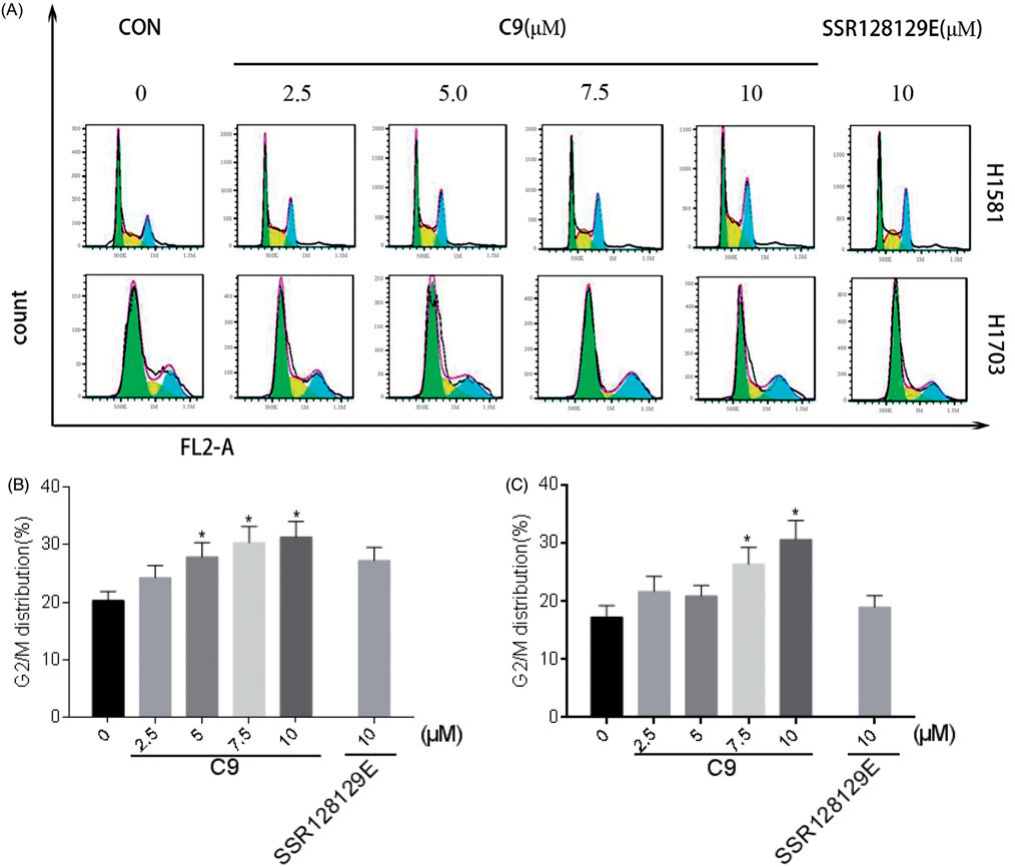
(A) Cell cycle inhibition of NCI-H1581 and NCI-H1703 cells treated with different concentrations of compound **C9** and 10 μM of SSR128129E control compound. (B) Cell cycle data analysis of NCI-H1581 cells treated with compound **C9**. (C) Cell cycle data analysis of NCI-H1703 cells treated with compound **C9** (*p < .05).

Cell cycle experiments were performed with NCI-H1581 and NCI-H1703 cells treated with 2.5 µM, 5.0 µM, 7.5 µM and 10 µM of **C9** and 10 µM SSR128129E, respectively. The data are presented in Figure 7, and demonstrate that 2.5 µM of **C9** significantly increased the number of NCI-H1581 cells in the G2 phase in a dose-dependent manner. Moreover, the effect of 10 µM of **C9** was better compared to that of SSR128129E. In addition, 7.5 µM of **C9** significantly increased the number of NCI-H1703 cells in the G2 phase at a dose-dependent manner. Moreover, the effect of 10 µM of **C9** was better than that of SSR128129E. Based on the results, we concluded that **C9** arrested cells with FGFR1 amplification in the G2 phase.

#### Kinase inhibitory activity and kinase selectivity profile

Selected tyrosine kinase receptors, such as FGFR1, EGFR, SRC and RET and ATP concentrations at Km were treated with 10 µM. Compound **C9** and inhibition rates were determined using a mobility shift assay (Table 3). The FGFR1 inhibition rate of **C9** was 84.3%, and the inhibition rates of other RTK receptors were lower, including wild-type and mutant EGFR family receptors, FLT family receptors, Janus kinase (JAK) family receptors. The relative maximum inhibition rate of other kinases was only 33.7%. Therefore, our data showed that **C9** is a selective FGFR1 inhibitor.

**Table 3.** Kinase inhibition rate after treatment with 10 μM of **C9**.

Kinase receptors	Inhibition rate (%)	Kinase receptors	Inhibition rate (%)
FGFR1	84.3	AKT1	16.0
FGFR2	23.6	CRAF	4.1
FGFR3	19.5	CKIT	23.5
FGFR4	15.3	AURA	30.4
EGFR	22.3	FLT1	7.6
HER2	0.5	FLT3	24.3
HER4	25.9	FLT4	15.1
EGFR L858R	11.1	JAK1	3.8
EGFR (d746-750)	33.7	JAK2	–3.6
EGFR T790M	9.8	RET	25.4
PDGFRα	5.2	TRK-A	32.2
PDGFRβ	11.8	IGF1R	10.6
SRC	16.5	IKKb	33.0
MET	31.3	TYK2	–9.3

#### Antiproliferative activity of compound C9 against a panel of intact cancer cell lines

To further illustrate the selectivity of compound **C9**, we selected four cells that do not express FGFR1 to determine the antiproliferative activity of compound **C9**. Seven concentrations (30, 20, 10, 1.0, 0.50, 0.10 and 0.01 µM) were set for the compound **C9**. Four EGFR addicted cell lines, including A549, PC-9, A431 and HCC827, were treated with various concentrations of the compound **C9**, respectively. The OD value was obtained and the inhibition rate was calculated, and the IC_50_ of compound **C9** on different cells was calculated by GraphPad Prism 5 software. The results obtained are presented in Table 4. Compared with the results of Table 2, it can be found that compound **C9** has better selectivity and activity on cells with high expression of FGFR1.

**Table 4.** Antiproliferative Effects of compound **C9** on EGFR addicted cell lines.

	Cell line			
	A549^a^	PC-9^b^	A431^c^	HCC827^d^
IC_50_ (μM)	29.63 ± 4.61	25.04 ± 4.38	>30	>30

a A549 is a human lung cancer cell line (WT EGFR/k-RAS dependent).

b PC-9 is a human lung cancer cell line (EGFR del E746_A750).

c A431 is a human epithelial carcinoma cell line (overexpressed WT EGFR).

d HCC827 is a human lung cancer cell line (EGFR dependent /WT k-RAS). The value “>30” indicates that no inhibitory effect at 30 μM compound concentration.

#### Molecular docking model of compound C9 and FGFR1

**Figure 8.**
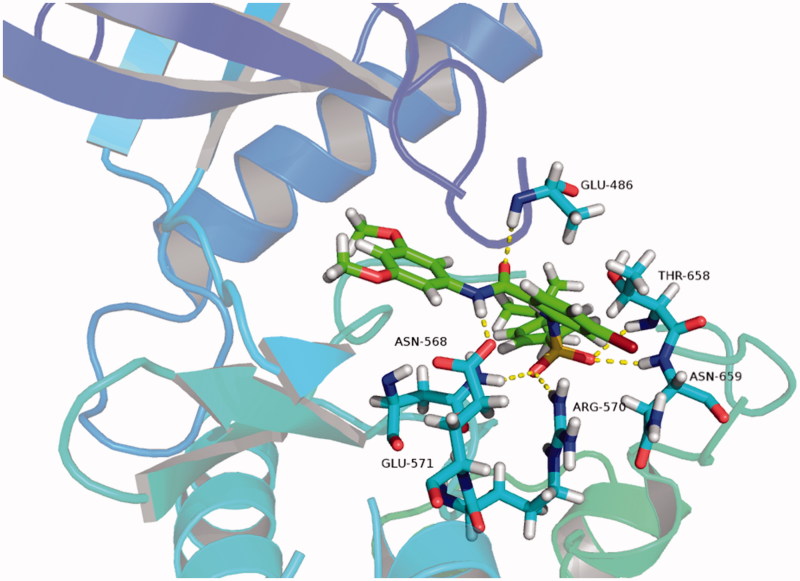
Molecular docking model of compound **C9** and FGFR1.

In this study, we used the Autodock software (version 4.2.6) to perform molecular docking experiments for compound **C9** and FGFR1 (PDB ID: 4V05), of which the results are presented in Figure 8. We found that compound **C9** formed four hydrogen bonds with amino acid residues Glu571, Arg570, Asn659 and Thr658 in the hydrophobic pocket 2, formed a hydrogen bond with amino acid residues Glu486 in the hinge region, and formed a hydrogen bond with amino acid residue Asn568 in the hydrophobic pocket 1. This experiment indicated several key hydrogen bonding interactions were formed, which may explain why compound **C9** has outstanding activity.

## Conclusion

In this study, we designed and synthesised 35 novel 4-bromo-N-(3,5-dimethoxyphenyl)benzamide derivatives, and evaluated the activity of these FGFR1-targeting compounds. Although most compounds demonstrated a modest inhibition of FGFR1, compound **C9** showed excellent inhibition of five NSCLC cell lines with FGFR1 amplification, which was significantly better than the lead compound SSR128129E. These findings may be due to the space structure of the FGFR1 ATP pocket, which was large enough to accommodate larger molecules and form more bonds. Although SSR128129E did not exhibit similar effects, compound **C9** significantly increased apoptosis in NCI-H226 and NCI-H1581 cell lines and in a dose-dependent manner. Cell cycle analysis showed that compound **C9** arrested NCI-H1581 and NCI-H1703 cell lines at the G2 phase. Furthermore, compound **C9** downregulated the phosphorylation of FGFR1, PLCγ1 and ERK in NCI-H226, NCI-H520 and NCI-H1581 cell lines. Based on *in vitro* enzymatic inhibitory activities of compound **C9** against different statuses of RTKs, and the antiproliferative activity of EGFR-addicted cells, it can preliminarily be concluded that compound **C9** is an FGFR1 inhibitor that has some degree of selectivity. Taken together, our results suggested that compound **C9** is a highly selective FGFR1 inhibitor with a novel chemical scaffold that may serve as a potential agent for further drug development in FGFR1-driven cancer therapy.

## Supplementary Material

IENZ_1460824_Supplementary_Material.pdf
