# Supplementary figures and images for: Design, synthesis and biological evaluation of 4-bromo-N-(3,5-dimethoxyphenyl)benzamide derivatives as novel FGFR1 inhibitors for treatment of non-small cell lung cancer
Source: J Enzyme Inhib Med Chem. 2018 May 7;33(1):905–19. doi: 10.1080/14756366.2018.1460824 (PMC6009922; doi:10.1080/14756366.2018.1460824)

## Supplementary Material

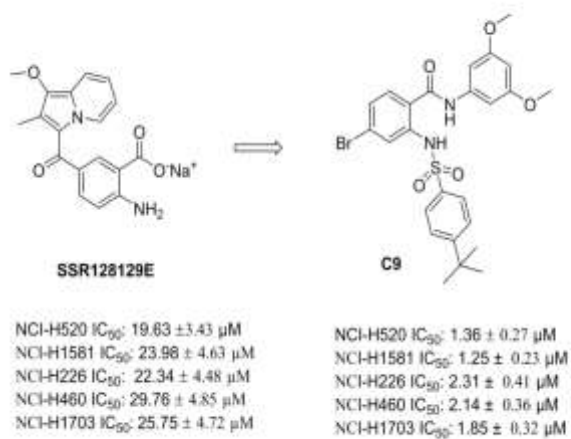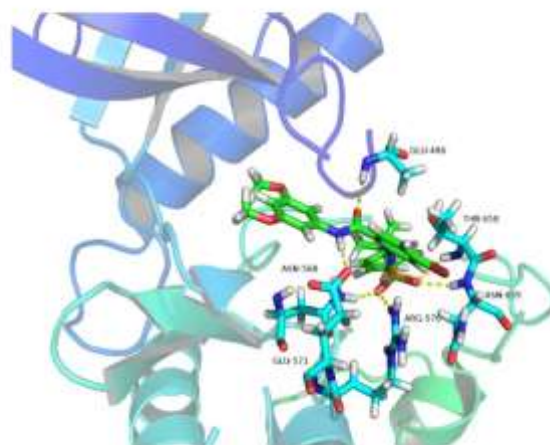

Supplement: IENZ_1460824_Supplementary_Material.pdf [file IENZ_A_1460824_SM4937.pdf]
